# Classification of healthcare-associated infection: a systematic review 10 years after the first proposal

**DOI:** 10.1186/1741-7015-12-40

**Published:** 2014-03-06

**Authors:** Teresa Cardoso, Mónica Almeida, N Deborah Friedman, Irene Aragão, Altamiro Costa-Pereira, António E Sarmento, Luís Azevedo

**Affiliations:** 1 Intensive Care Unit, Unidade de Cuidados Intensivos Polivalente, Hospital de Santo António, University of Porto, Largo Prof. Abel Salazar, 4099-001 Porto, Portugal; 2 Internal Medicine Department, Hospital de Braga, Sete Fontes - São Vitor, 4710-243 Braga, Portugal; 3 Department of Medicine, Barwon Health, Geelong, VIC 3220, Australia; 4 Department of Health Information and Decision Sciences, Center for Research in Health Technologies and Information Systems (CINTESIS), Faculty of Medicine, University of Porto, Alameda Prof. Hernâni Monteiro, 4200-319 Porto, Portugal; 5 Department of Infectious Diseases, Hospital de São João, University of Porto, Alameda Prof. Hernâni Monteiro, 4200-319 Porto, Portugal

**Keywords:** Healthcare-associated infection, Classification, Multidrug resistant pathogens prevalence, Pneumonia, Bloodstream infections, Endocarditis, Urinary tract infections, Intra-abdominal infections

## Abstract

**Background:**

Ten years after the first proposal, a consensus definition of healthcare-associated infection (HCAI) has not been reached, preventing the development of specific treatment recommendations. A systematic review of all definitions of HCAI used in clinical studies is made.

**Methods:**

The search strategy focused on an HCAI definition. MEDLINE, SCOPUS and ISI Web of Knowledge were searched for articles published from earliest achievable data until November 2012. Abstracts from scientific meetings were searched for relevant abstracts along with a manual search of references from reports, earlier reviews and retrieved studies.

**Results:**

The search retrieved 49,405 references: 15,311 were duplicates and 33,828 were excluded based on title and abstract. Of the remaining 266, 43 met the inclusion criteria. The definition more frequently used was the initial proposed in 2002 - an infection present at hospital admission or within 48 hours of admission in patients that fulfilled any of the following criteria: received intravenous therapy at home, wound care or specialized nursing care in the previous 30 days; attended a hospital or hemodialysis clinic or received intravenous chemotherapy in the previous 30 days; were hospitalized in an acute care hospital for ≥2 days in the previous 90 days, resided in a nursing home or long-term care facility. Additional criteria founded in other studies were: immunosuppression, active or metastatic cancer, previous radiation therapy, transfer from another care facility, elderly or physically disabled persons who need healthcare, previous submission to invasive procedures, surgery performed in the last 180 days, family member with a multi-drug resistant microorganism and recent treatment with antibiotics.

**Conclusions:**

Based on the evidence gathered we conclude that the definition initially proposed is widely accepted. In a future revision, recent invasive procedures, hospitalization in the last year or previous antibiotic treatment should be considered for inclusion in the definition. The role of immunosuppression in the definition of HCAI still requires ongoing discussion.

## Background

Traditionally, infections have been classified as community or hospital-acquired, according to their place of acquisition, and this classification is still used to guide treatment decisions [1,2].

Over the last decade the massive increase in outpatient clinical care has led to a new context for the emergence of healthcare-associated infections (HCAI). This is a new name for a new group of infections emerging among patients that come from the community with a history of previous exposure to healthcare who do not fit the nosocomial infection criteria. The proportion of patients hospitalized with HCAI among those admitted from the community setting can be as high as 50% [3-6].

The first proposals of HCAI and its inclusion in infection classification along with community-acquired infection (CAI) and hospital-acquired infection (HAI) were made in 2002 by Siegman-Igra et al. [7] and Friedman et al. [3]. Different one from another, the definition from Friedman et al. [3] has been used in numerous clinical studies and will be referred to in this review as the initial definition; it is defined as an infection present at hospital admission or within 48 hours of admission in patients that fulfilled any of the following criteria:

– received intravenous therapy at home, wound care or specialized nursing care through a healthcare agency, family or friends; or had self-administered intravenous medical therapy in the 30 days before the infection;

– attended a hospital or hemodialysis clinic or received intravenous chemotherapy in the previous 30 days;

– were hospitalized in an acute care hospital for 2 or more days in the previous 90 days,

– resided in a nursing home or long-term care facility.

Although widely accepted [5,8-10] numerous alternative definitions have also been used in clinical studies [11-14]. This heterogeneity has raised more confusion than understanding in determining likely microbiological resistance patterns and making decisions about empiric antibiotic treatment. A correct recognition of all risk factors for HCAI is crucial in guaranteeing optimal empiric antibiotic choice to adequately treat likely pathogens while avoiding selective pressure that contributes to the development of multidrug-resistant (MDR) organisms.

The objective of the current study is to present a systematic review of all definitions of HCAI used in clinical studies in order to compare and contrast the criteria they include.

## Methods

### Data sources and searches

**Figure 1:**
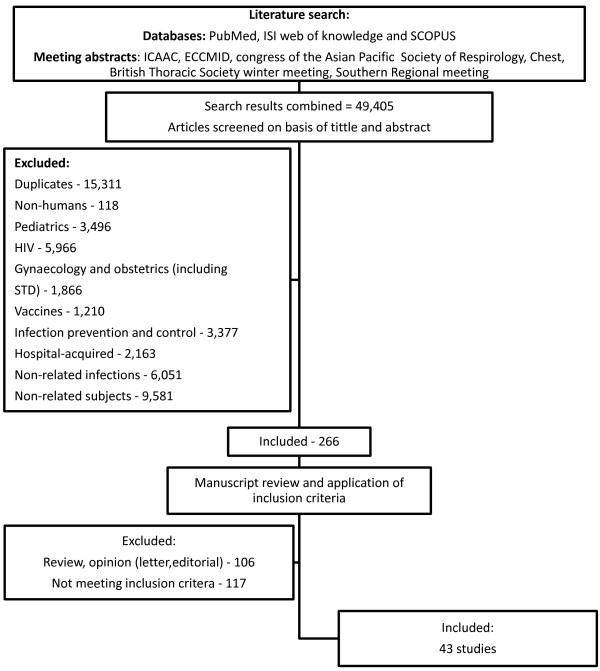
Flow diagram of study selection.

This search was performed in accordance with the recommendations of the Cochrane collaboration using MEDLINE/PubMed, SCOPUS and ISI Web of Knowledge from the earliest achievable data until November 2012. A manual search of references from reports, earlier reviews and retrieved studies was also performed. Abstract books and CD-ROMs from several annual scientific meetings were searched for relevant abstracts (Figure 1). No language restriction was applied and papers written in a foreign language were translated.

The electronic search strategy covered the main subject area: healthcare-associated infection (Additional file 1: Search strategy details). The last search was done on 8 November 2012.

### Study selection

The inclusion criteria were all observational studies (cohort, cross-sectional or case–control) on adult patients admitted to hospital that provided microbiology results according to place of acquisition of infection. The following definitions of infection by setting were used:

– CAI - infection detected within 48 hours of hospital admission in patients without previous contact with healthcare service.

– HAI - localized or systemic condition: 1) that results from adverse reaction to the presence of an infectious agent(s) or its toxin(s) and 2) that was present 48 hours or more after hospital admission and not incubating at hospital admission time [15].

– HCAI - infection detected within 48 hours of hospital admission in patients that had previous contact with healthcare service within one year.

### Data extraction and quality assessment

The results of the literature search were accessed by two reviewers (TC, MA) and non-relevant studies were excluded based on title and abstract. For potentially relevant studies, the full text was obtained, and two investigators (TC, MA) independently assessed study eligibility and extracted data on study design, objectives, HCAI definitions and multi-drug resistant pathogens (MDR) prevalence (Methicillin-resistant Staphylococcus aureus (MRSA), Pseudomonas aeruginosa, Acinectobacter, Stenotrophomas maltophilia, extended-spectrum beta lactamases producer (ESBL)), using a data extraction protocol; disagreements were resolved through consultation with a third reviewer (LA).

Each selected study was independently evaluated by two reviewers (TC, MA) for the strength of evidence through examination of the study design and quality of data.

Potential threats to the internal validity of included studies were evaluated considering the following criteria:

– The authors define inclusion criteria,

– The authors define an adequate selection method,

– The selection of participants was consecutive,

– The outcome data (microbiology data by place of acquisition) were complete and reported (no attrition bias) and

– All results were reported (reporting bias).

Studies that met all of the above five criteria, were classified as “low risk of bias”. Studies that partially met one or more criteria were classified as “moderate risk of bias”. Studies were classified as “high risk of bias” if one or more of these criteria was not met.

### Data analysis

Data on individual studies included are provided in Tables 1 and 2. A meta-analysis was not performed due to the nature of the objectives of this review and the heterogeneity of the studies included.

**Table 1:** **Characterization of included studies that use the initial definition of HCAI**[3]**by focus of infection**

**Author, publication year**	**Study design**	MDR* organisms, n (%)			**Risk of bias**
**Bacteriemia**		**CAI**	**HCAI**	**HAI**	
Friedman *et al.*, 2002 [3]	Prospective, multicenter, 504 patients, USA, 4 to 5 months	Not mentioned			High
Marschall *et al.*, 2009 [16]	Prospective, single center, 250 patients, Gram-negative, USA, 6 months	0 (0)	9 (7)	10 (11)	Low
Evans *et al.*, 2009 [17]	Retrospective, multicenter, 223 patients, spinal cord injury, USA, 7 years	6 (17)	34 (31)	111 (42)	High
Son *et al.*, 2010 [18]	Prospective, multicenter, 1,144 patients, Korea, 12 months	29 (8)	38 (7)	162 (79)	Low
Rodriguez-Bano *et al.*, 2010 [19]	Prospective, multicenter, 821 bacteremia episodes including potential contaminants, Spain, 2 to 5 months	7 (5)	29 (15)	99 (21)	Moderate
Vallés *et al.*, 2011 [8]	Prospective, multicenter, 726 patients, Spain and Argentina, 12 months	7 (2)	11 (8)	29 (12)	High
**Pneumonia**		**CAI**	**HCAI**		
Carratalà *et al.*, 2007 [10]	Prospective, single center, 727 patients, those with neutropenia, AIDS and after transplantation were excluded, Spain, 4 years.	3 (1)	2 (2)		Low
Shindo *et al.*, 2009 [20]	Retrospective, single center, 371 patients, Japan, 1 year and 3 months	6 (6)	17 (22)		Low
Park *et al.*, 2010 [9]	Retrospective, single center, 345 patients, CAI and HCAI were considered until 72 h after hospital admission; patients with neutropenia, AIDS and after transplantation were excluded, Korea, 1 year.	7 (15)	21 (32)		Moderate
Pascual *et al.*, 2010 [21]	Retrospective, single center, 308 patients with bacteriemic pneumonia, Spain, 6 years	CAI (2)	HCAI (12)	HAI (31)	High
Umeki *et al.*, 2011 [22]	Prospective, single center, 202 patients, Japan, 2 years	10 (21)	12 (25)		Moderate
Seki *et al.*, 2011 [23]	Retrospective, single center, 34 patients Japan, 4 months	0 (0)	6 (43)		Moderate
Garcia-Vidal *et al.*, 2011 [24]	Prospective, single center, 2,153 patients, those with more than one condition of HCAI, with neutropenia, AIDS, after transplantation and chronic corticosteroid treatment were excluded, Spain, 8 years and 9 months	19 (2)	7 (2)		Low
Jung *et al.*, 2011 [25]	Retrospective, single center, 527 patients, Korea, 1 year	15 (18)	30 (38)		Low
Jeon *et al.*, 2011 [26]	Retrospective, multicenter, 210 patients older than 60 years, Korea, 2 years.	10 (16)	20 (67)		Low
Depuydt *et al.* 2011 [27]	Retrospective, single center, 269 patients, those with neutropenia, transplantation or transferred from another hospital were excluded, Belgium, 1 year	0 (0)	6 (30)		Low
Park *et al.*, 2012 [4]	Prospective, single center, 339 patients, Korea, 2 years	35 (20)	52 (31)		Low
Lee *et al.*, 2012 [28]	Retrospective, multicenter study, 250 patients, Korea, 21 months	3 (4)	17 (31)		Low
**Other foci**		**CAI**	**HCAI**	**HAI**	
Benito *et al.*, 2009 [5]	Prospective, single center,1622 patients with endocarditis, comparing CAI with healthcare associated that included non-nosocomial and nosocomial, intra-venous drug users and prosthetic valves were excluded, USA, 6 months.	25 (3)	41 (17)	76 (26)	Low
Wu *et al.*, 2011 [29]	Retrospective, single center,192 patients with endocarditis, Taiwan, 5 years	15 (11)	13 (43)	17 (81)	Moderate
Aguilar-Duran *et al.*, 2012 [6]	Prospective, single center, 251 patients with urinary infection, Spain, 7 to 8 months	2 (2)	15 (15)	9 (14)	High

*MDR, MRSA, Pseudomonas sp, Acinectobacter, Stenotrophomonas maltophilia, ESBL. CAI, community-acquired infection; HAI, hospital-acquired infection; HCAI, healthcare-associated infection; MDR, multidrug-resistant.

**Table 2:** **Characterization of included studies that did not use the initial definition of HCAI**[3]**by focus of infection**

**Study, year of publication**	**Study design**	**HCAI criteria**	MDR* organisms			**Risk of bias**
**A. Bloodstream infections**						
Siegman-Igra *et al.*, 2002 [7]	Prospective, single center, 1,028 infections in 912 patients; Israel, 1 year	1. Discharge from hospital 2 to 30 days previously	CAI	HCAI	HAI	Moderate
		2. Nursing-home acquired	8 (2)	24 (10)	71 (17)	
		3. Patients with long-term intravenous devices, for hemodialysis, chemotherapy or parenteral nutrition				
		4. Chronic hemodialysis				
		5. Invasive procedure previously or at hospital admission				
Shorr *et al.*, 2006 [11]	Retrospective, multicenter, 6,697 patients; USA, 2 years	1. Prior hospitalization within 30 days	CAI	HCAI	HAI	Low
		2. Transfer from another healthcare facility	152 (2)	397 (11)	62 (13)	
		3. Chronic hemodialysis				
		4. Immunosuppression medication or metastatic cancer				
Kao *et al.*, 2011 [30]	Prospective, single center, 890 infections in 831 patients older than 14 years; Taiwan, 1 year	1. Hospitalized for 2 or more days in the previous 90 days	CAI	HCAI	HAI	High
		2. Resided in a nursing home	11 (2)	41 (16)	42 (41)	
		3. Hemodialysis, intravenous chemotherapy or invasive procedures in the previous 90 days				
Kollef *et al.*, 2011 [12]	Prospective, multicenter, 1,143 patients; USA, 1 year	1. Prior hospitalization within 6 months	CAI	HCAI		Low
		2. Admission from a skilled nursing facility	79 (19)	242 (33)		
		3. Hemodialysis				
		4. Immunosuppression				
Al-Hasan *et al.*, 2012 [31]	Retrospective, multicenter, 733 episodes of gram negative bacteremia; excludes polymicrobial, nosocomial and recurrent episodes; USA, 10 years	1. Hospitalized for 2 or more days in the previous 90 days	CAI	HCAI		Moderate
		2. Residents in a nursing home or long-term care facility	12	28 (7)		
		3. Received intravenous therapy including ATB and chemotherapy				
		4. Hemodialysis in the previous 30 days				
Lenz *et al.*, 2012 [32]	Retrospective, multicenter, 7,712 patients; only samples obtained in the first 5 days of hospital admission; Canada, 8 years	1. Hospitalized for 2 or more days in the previous 90 days	CAI	HCAI	HAI	Low
		2. Nursing home or long term-care facility	59 (2)	158 (6)	202 (9)	
		3. Visit a hospital, clinic or emergency department within the prior 5 to 30 days				
		4. Hemodialysis				
		5. Active cancer				
**B. Pneumonia**						
Kollef *et al.*, 2006 [13]	Retrospective, multicenter, 4,543 patients with positive cultures within 5 days of hospital admission, USA, 2 years	1. Prior hospitalization within 30 days	CAI	HCAI	HAI	High
		2. Admission from another care facility	624 (28)	537 (54)	362 (43)	
		3. Receiving long-term hemodialysis				
Micek *et al.*, 2007 [33]	Retrospective, single center, 639 patients older than 16 years with positive cultures, Spain, 2 years	1. Hospitalization in the past 12 months	CAI	HCAI		Low
		2. Resident in a nursing home or long-term care facility or rehabilitation hospital	35 (17)	242 (56)		
		3. Outpatient hemodialysis, peritoneal dialysis or infusion therapies requiring regular visits to a clinic				
		4. Immunosuppression				
Schreiber *et al.*, 2010 [34]	Retrospective, single center study, 190 patients needing mechanical ventilation more than 24 hours after hospital admission, patients without evidence of bacterial infection and patients transferred from other hospitals were excluded, USA, 4 years	1. Recent hospitalization (90 days)	CAI	HCAI		Low
		2. Admission from a long-term facility	17 (18)	48 (48)		
		3. Recent treatment with broad spectrum antibiotics (30 days)				
		4. Chronic hemodialysis				
		5. Wound care				
		6. Immunosuppression				
Grenier *et al.*, 2011 [14]	Retrospective, single center study, 3,295 patients, those transferred from other hospitals or discharged from an acute care facility within 14 days were excluded, Canada, 12 years	1. Hospitalization in the past 90 days, but not in the last 14 days	CAI	HCAI		Low
		2. Resident of a nursing home or long-term care facility	42 (4)	21 (10)		
		3. Outpatient intravenous therapy or cancer therapy within the previous month				
		4. Long term hemodialysis				
Guimarães *et al.*, 2011 [35]	Retrospective, single center study, 197 patients, Portugal, 1 year	1. Hospitalization for 2 days or more in the preceding 90 days	CAI	HCAI		High
		2. Resident of a nursing home or extended-care facility	2 (18)	9 (82)		
		3. Intravenous antibiotic therapy or chemotherapy within 30 days				
		4. Chronic dialysis within 30 days				
		5. Home wound care				
		6. Family member with multi-drug resistant pathogen				
Chalmers *et al.*, 2011 [36]	Prospective, single center, 1,348 patients; Scotland, 4 years and 5 months	1. Hospitalization for 2 days or more in the preceding 90 days	CAI	HCAI		High
		2. Resident of a nursing home or extended-care facility	3 (1)	5 (6)		
		3. Home infusion therapy (including antibiotics and long-indwelling devices as catheters)				
		4. Chronic dialysis within 30 days				
		5. Home wound care				
		6. Family member with multi-drug resistant pathogen				
Tasbakan *et al.*, 2011 [37]	Retrospective, single center study, 187 patients, Turkey, 1 year	1. Hospitalization for 2 days or more in the preceding 90 days	CAI	HCAI		Low
		2. Resident of a nursing home or extended-care facility	1 (13)	17 (44)		
		3. Home infusion therapy (including antibiotics)				
		4. Chronic dialysis within 30 days				
		5. Home wound care				
		6. Family member with multi-drug resistant pathogen				
Ishida *et al.*, 2012 [38]	Retrospective, single center, 893 patients; Japan, 3 years	1. Discharged from a hospital in the preceding 90 days	CAI	HCAI		Low
		2. Resident in a nursing home or extended-care ward	6 (3)	37 (21)		
		3. A patient who regularly requires vascular access for dialysis, antimicrobial treatment, chemotherapy or immunosuppressive therapy in an outpatient setting				
		4. Elderly or handicap who needs long-term care with an ECOG of 3 or 4				
Miyashita *et al.*, 2012 [39]	Prospective, multicenter, 1,385 patients; CAI and HCAI were considered until 72 h after hospital admission, Japan, 6 years	1. Discharged from a hospital in the preceding 90 days	CAI	HCAI		High
		2. Resident in a long-term nursing home setting or healthcare home	21 (4)	78 (20)		
		3. Continuous receiving endovascular therapy in an ambulatory setting (including dialysis, antibiotics, anticancer drugs and immunosuppression)				
		4. Elderly persons or physical disable persons who need healthcare				
Wu *et al.*, 2012 [40]	Retrospective, multicenter study, 1,646 patients, those with pneumonia developing 2 days after admission or less than 14 days after the last hospitalization, with lung cancer obstructive pneumonia or HIV positive status with CD4+ t <200 were excluded, Taiwan,1 year	1. Undergoing repeated hospitalization within 90 days before	CAI	HCAI		Low
		2. Residing in a nursing home	122 (15)	169 (28)		
		3. Receiving radiation therapy or chemotherapy at an outpatient clinic				
		4. Received regular dialysis at an out patients clinic				
Sugisaki *et al.*, 2012 [41]	Retrospective, single center study, 526 patients, Japan, 4 years	1. Hospitalization for 2 days or more in the preceding 90 days	CAI	HCAI		Low
		2. Resident of a nursing home or extended-care facility	50 (15)	72 (40)		
		3. Home infusion therapy (including antibiotics and long indwelling devices as catheters)				
		4. Chronic dialysis within 30 days				
		5. Home wound care				
		6. Family member with multi-drug resistant pathogen				
Giannella *et al.*, 2012 [42]	Prospective, multicenter, 1,002 patients older than 16 years admitted into internal medicine departments, Spain, 2 weeks	1. Hospitalization in the past 180 days	CAI	HCAI	HAI	Low
		2. Resident in a nursing home or extended-care facility	6 (4)	19 (29)	11 (52)	
		3. Attending a hospital regularly because of chronic underlying disease				
		4. Undergoing hemodialysis				
		5. Wound care or specialized nursing care in the past 30 days				
		6. Chemotherapy in the past 30 days				
		7. Surgery in the past 180 days				
**C. Other foci**						
Swenson *et al.*, 2009 [43]	Retrospective, single center study, 2,049 intra-abdominal infections; Canada, 10 year	1. Patients with a story of any hospitalization in the previous 30 days	CAI	HCAI		High
		2. Resident of a nursing home or rehabilitation facility in the previous 30 days	27 (7)	221 (28)		
Merli *et al.*, 2010 [44]	Prospective, single center study, in 54 patients with cirrhosis, patients with HIV infection, under high dose of corticosteroid treatment or immunosuppressive therapy were excluded, Italy, 9 months	1. Hospitalization for 2 or more days or had undergone surgery during the preceding 180 days	CAI	HCAI	HAI	Low
		2. Resident of a nursing home or long-term care facility	2 (50)	9 (82)	5 (45)	
		3. Attended a hospital or hemodialysis clinic or received intravenous chemotherapy in the 30 days before				
Ha *et al.*, 2011 [45]	Retrospective, single center study, in 319 patients with urinary infection; Korea, 1 year	1. Hospitalization for 2 or more days in an acute care hospital in the preceding 90 days	CAI	HCAI		Low
		2. Resident of a nursing home or long-term care facility	1 (1)	28 (14)		
		3. Received intravenous therapy, wound care or specialized nursing care at home in the previous 30 days				
		4. Attended a hospital or hemodialysis clinic or received intravenous chemotherapy in the 30 days before				
		5. Received an invasive procedure, urological surgery or urethral catheterization in the previous 7 days				
Sy *et al.*, 2012 [46]	Retrospective, multicenter study, in 1,536 patients with endocarditis; patients transferred from another hospital, recurrent admissions or day-stay admissions were excluded, Australia, 6 years	1. Hospitalization for 2 days or more in the preceding 90 days	HCAI	Non-HCAI		High
		2. Resident of a nursing home or in a long-term facility	40 (26)	61 (18)		
		3. Attended a hemodialysis clinic or received intravenous therapy in the 30 days before				

*MDR = MRSA, Pseudomonas sp, Acinectobacter, Stenotrophomonas maltophilia, ESBL. CAI, community-acquired infection; ECOG, electrocochleography; HAI, hospital-acquired infection; HCAI, healthcare-associated infection; MDR, multidrug-resistant.

## Results

The search retrieved a total of 49,405 references. Of the 266 studies included in the first review, 106 were review articles or opinion pieces and 117 did not meet inclusion criteria. Of the remaining 52 studies: 30 used the initial definition of Friedman et al. [3], but only 21 provided data on microbiology and were included along with 22 additional studies that used alternative definitions and met the inclusion criteria. Of the 43 studies included in this systematic review (Figure 1): 18 were prospective (7 multicenter and 11 single center) and 25 were retrospective (9 multicenter and 16 single center); involving 42,611 patients.

Characteristics of included studies that used the initial definition are shown in Table 1 and of those that used alternative definitions in Table 2.

### Infections by source

In bloodstream HCAIs, six studies used the initial definition [3] (Table 1) and six did not (Table 2), all found an increasing prevalence of MDR organisms from CAI to HCAI and HAI, regardless of the definition used.

The majority of the included studies were about pneumonia (24 studies of 43). Most of these studies only compared community-acquired pneumonia (CAP) with healthcare-associated pneumonia (HCAP) and revealed a higher prevalence of MDR pathogens among HCAP patients compared with CAP patients. There were three studies comparing CAP and HCAP with hospital acquired pneumonia (HAP) [13,21,42] but they used different definitions of HCAP achieving different results regarding MDR prevalence according to place of acquisition of infection (Tables 1 and 2).

There were three studies of healthcare-associated infective endocarditis [5,29,46]. Two found an increasing rate of MDR organisms from community-acquired to healthcare-associated and hospital-acquired infective endocarditis (Table 1). A third study compared healthcare-associated infective endocarditis with non-healthcare-associated infective endocarditis (that is, community-acquired plus hospital-acquired infective endocarditis) and found a higher prevalence of MDR in healthcare-associated infective endocarditis than in non-healthcare-associated infective endocarditis (Table 2).

Studies regarding urinary tract [6,45] and intra-abdominal [43] infections also found a higher prevalence of MDR organisms, among HCAIs when compared to CAIs (Tables 1 and 2).

The initial definition [3] was the most widely used in clinical studies (30 studies among 52). Overall, different HCAI definitions comprised 17 different criteria, of which 7 were equivalent to those used by Friedman et al. [3], but leading to a different final definition due to the addition or subtraction of criteria (Table 3).

**Table 3:** List of all different criteria used to compose different classifications of HCAI

**Criteria for HCAI**	**Low risk of bias**	**Moderate risk of bias**	**High risk of bias**	**All studies**
**Criteria included in the initial definition [**[3]**]**	**Number of studies (number of patients)**			
Received intravenous therapy	16 (13,082)	7 (3,239)	8 (6,284)	31 (22,605)
Received wound care or specialized nursing care	16 (10,086)	5 (1,594)	7 (3,560)	28 (15,240)
Attended a hospital or a clinic in the last 30 days	16 (17,588)	5 (1,594)	5 (2,015)	26 (21,197)
Received chemotherapy in the last 30 days	15 (10,883)	6 (2,506)	7 (3,043)	28 (16,432)
Receiving hemodialysis	23 (31,272)	7 (2,639)	10 (10,470)	40 (44,381)
Prior hospitalization (in the last year)	24 (32,165)	7 (3,239)	12 (13,904)	43 (49,308)
Resident in a nursing home or long term-care facility	23 (25,468)	7 (3,239)	11 (9,361)	41 (38,068)
**Criteria NOT included in the initial definition**[3]				
Transfer from another care facility	1 (6,697)		1 (4,543)	2 (11,240)
Immunosuppression	4 (8,669)			4 (8,669)
Active or metastatic cancer	1 (7,712)			1 (7,712)
Submitted to invasive procedures previously	1 (319)	1 (912)	1 (831)	3 (2,062)
Family member with a multi-drug resistant microorganism	2 (713)		2 (1,545)	4 (2,258)
Elderly person or physical disable persons who need healthcare	1 (893)		1 (1,385)	2 (2,278)
Surgery in the last 180 days	2 (1,056)			2 (1,056)
Received radiation therapy	1 (1,646)			1 (1,646)
Recent (30 days) treatment with antibiotics	1 (190)	1 (733)	1 (197)	3 (1,120)

HCAI, healthcare-associated infection.

An analysis of the risk of bias of the 43 included studies revealed that 24 presented a low risk of bias, 7 presented a moderate risk of bias, and 12 presented a high risk of bias, according to previously defined criteria (Additional file 2: eTable 1 - Studies with moderate or high risk of bias according to pre-defined criteria).

## Discussion

Ten years after the first descriptions [3,7], this is the first systematic review of HCAI classification. It incorporates all published studies on HCAI that provided original data. The majority of the included studies had a low risk of bias, resulting in good quality of the evidence assembled.

The following criteria that were used in various studies to define HCAI in patients with an infection present at hospital admission or within 48 hours of admission are the ones that we believe to be most important:

– received invasive procedures in the 30 days before the infection, including specialized nursing care;

– attended a hospital or hemodialysis clinic in the previous 30 days;

– were hospitalized in an acute care hospital for 2 or more days in the previous year;

– resided in a nursing home or long-term care facility;

– treatment with broad spectrum antibiotics in the last 30 days.

The initial HCAI definition [3] included treatments delivered at home or in an outpatient clinic and these criteria have been widely adopted in other studies. The receipt of intravenous therapy [7,14,30,31,35-39,41,45-47], wound care or specialized nursing care [34-37,41,42,45], and hemodialysis [7,11-14,31-42,44-46], as well as attendance at a hospital or clinic [32,42,44,45] are important factors as this group of patients has documented higher rates of colonization and infection with MDR microorganisms [48-50]. Three additional studies have included the criteria of other previous invasive procedures [7,30,45], like urological procedures [45]. There is no reason to believe that this last group of patients is different from the previous ones in regards to the risks of infection by MDR organisms, so we propose that the first criterion be generalized to include all patients that received invasive procedures in the 30 days before the infection.

The second criterion in the initial definition [3] includes receiving chemotherapy in the last 30 days. This is a criterion frequently used among alternative definitions [7,14,30,31,35,38-40,42,44],[45] along with having active or metastatic cancer [11,32] that suggest receipt of some kind of anti-cancer therapy. These are a special group of patients due to underlying immunosuppression. Immunosuppression, including HIV infection and treatment with immunosuppressive agents is a criterion considered by some authors [11,12,33,34,38,39], but specifically excluded by others [40,44]. The variety of potential opportunistic pathogens that may occur among this group of patients varies largely according to the underlying cause of immunosuppression, for example empiric antimicrobial recommendations for a patient with advanced HIV infection [51] are distinct from therapies used in patients with acute febrile neutropenia [52]. The inclusion of these groups of patients in a HCAI definition is possibly one of the most controversial issues and for the moment we suggest that they be excluded from the definition, supported by the existence of specific recommendations for these special populations.

Nevertheless, many immunosuppressed patients, including cancer patients would fulfill other criteria for HCAI, such as invasive procedures, recently attending a hospital clinic, recent hospitalization and/or recent treatment with broad spectrum antibiotics.

Regarding previous hospitalization, we believe that this criterion must be retained in any definition of HCAI. The presence of MDR organisms (gram positives or gram negatives) has been documented between six months to one year after hospital discharge [53-55]. This risk of long lasting colonization of both the respiratory tract and gastrointestinal tract with pathogens not present in the community following hospitalization has led some authors to alter this criterion to hospitalization in the previous six months [12,42,44] or even one year [33]. However, the classification of infections that develop among patients recently discharged from the hospital (in the previous 14 days) is somewhat contentious. Some authors consider these infections occurring within 14 days of hospital discharge nosocomial infections [48,50,56], while others consider infections among those hospitalized in the last month as HCAIs [11,14]. Based on the existing evidence, we propose that in the third criterion time from the last hospitalization will be enlarged to one year and patients discharged from the hospital within the last two weeks be considered as having a hospital-acquired infection.

Patients admitted from nursing homes with infection have been extensively studied and may constitute more than 50% of cases of healthcare-associated pneumonia [49]. This criterion has been considered by almost all studies; however, caution is needed with this approach. Patients with non-severe nursing home-acquired pneumonia (NHAP) have a pathogen distribution similar to those expected in CAP [57]. Among patients with severe NHAP, with organ dysfunction, resistant pathogens have been seen [10,36,57]. Poor functional status and increased age have been linked to an increased risk of infection with a MDR pathogen among NHAP patients [20,58], and are linked to the level of care provided in these facilities. Nursing homes with hospital-like wards carry the same infection risk by resistant pathogens as hospitals, and should best be considered as the analogous to HAIs. Clinicians should consider factors such as functional status and level of care required in selecting treatments for patients who reside in nursing homes.

Recent treatment with broad spectrum antibiotics has been identified as a risk factor for infection or colonization by MDR pathogens [59] and should also be considered both in the definition of HCAI and in selection of empiric antibiotics.

Patients with close contact with a family member with a MDR microorganism are part of the American Thoracic Society (ATS) definition of HCAP [2]. Currently, there are no epidemiological studies assessing the microbiological features of this particular group of patients [50] sustaining its inclusion on the HCAI definition.

Additional criteria not included in the initial definition [3] represent different descriptions of the same criteria: active or metastatic cancer, submission to invasive procedures or transfer from another care facility.

There has only been one previous review on HCAI to our knowledge. It concerns healthcare-associated pneumonia and is focused mainly on epidemiology [60]. The authors performed the search in PubMed, and included eight studies regardless of the definition used. No assessment of bias was made. A description of the definitions of HCAP used was not made. Five of those studies focused only on nursing-home acquired pneumonia. The remaining three studies of HCAP included by the authors were also included in the current analysis. Recently, new definitions of Lab-ID infections were published [61] based on laboratory testing data without a clinical evaluation of the patient, allowing colonization to be counted as infection. Nevertheless, this methodology might facilitate surveillance of multi-drug resistant organisms (MDROs) among patients in the outpatient clinic and long-term care facilities and nursing home settings. Of notice is the fact that the document categorizes MDRO LabID events in: community-onset if the specimen was collected as an outpatient or inpatient three or more days after admission and healthcare facility-onset if the LabID event specimen was collected more than three days after admission to the facility. Following this definition, a patient with HCAI is included in community-onset LabID event, representing from our point of view a major step backward in the classification of infection according to place of acquisition.

This systematic review provides the clinician with a thorough description of all criteria available in order to include an infected patient in the category of HCAI, in the hope that it leads to an optimal selection of empiric antibiotic therapy in this group of patients and consequently an improvement in outcome. It is expected that a consensus definition of HCAI can be developed to be used in future research in order to develop specific antibiotic recommendations for this group of patients.

The future definition of HCAI should be universal regardless of the focus of infection if its use is intended to be immediate at the bedside like it happened with the classic dichotomy classification of infection in community and hospital-acquired infection, which allowed the prompt institution of adequate antibiotic therapy, a major prognostic factor. Nevertheless, specific risk factors for infection by a particular microorganism should always be taken into account by the clinician.

### Strengths and limitations

Despite the extensive research done, including electronic search in several databases, relevant conference proceedings and a hand search of additional sources, there is always the possibility of missing studies that could meet the inclusion criteria.

Researcher bias is always a possibility in this type of analysis; in order to reduce it we had two independent researchers review the articles and a third one resolve disagreements along with strict and simple inclusion criteria established prior to the research.

The permissive criteria for inclusion in this study were essential to achieve the main goal: gathering all definitions of HCAI used in clinical studies.

We found a high rate of studies with low risk of bias, probably related to the simplicity of the evaluation. Considering that we only found observational studies we think that the criteria adopted were the most adequate to evaluate risk of bias in this type of studies.

## Conclusions and recommendations

The initial definition of HCAI [3] seems to be widely accepted. Some of the included criteria, such as attendance at a hospital or hemodialysis clinic in the previous 30 days and residence in a nursing home or long-term care facility should be maintained; the precise time from the last hospitalization is still controversial and probably should be extended to one year. Additional criteria as recent invasive procedures and receipt of broad-spectrum antibiotics should be considered for inclusion in a future definition of HCAI.

The inclusion/exclusion of immunosuppressed patients in the definition of HCAI requires ongoing discussion.

It is expected that a consensus definition of HCAI can be developed soon to be used in future research in order to develop specific antibiotic recommendations for this group of patients, with an influence from local antibiograms.

## Abbreviations

ATS: American Thoracic Society; CAI: Community- acquired infection; CAP: Community-acquired pneumonia; ESBL: Extended-spectrum beta lactamases producer; HAI: Hospital-acquired infection; HAP: Hospital acquired pneumonia; HCAI: Healthcare-associated infection; HCAP: Healthcare-associated pneumonia; HIV: Human immunodeficiency virus; MDR: Multidrug-resistant; MDRO: multi-drug resistant organism; MRSA: Methicillin-resistant Staphylococcus aureus; NHAP: Nursing home-acquired pneumonia

## Competing interests

The authors declare that they do not have any competing interests.

## Authors' contributions

TC, MA, LA, IA, ACP and AS were responsible for study concept and design. TC and MA acquired the data. TC, MA, LA and NDF were responsible for analysis and interpretation of data and drafting of the manuscript. All the authors took part in the revision of the manuscript and approval of the final version to be published.

## Pre-publication history

The pre-publication history for this paper can be accessed here:

http://www.biomedcentral.com/1741-7015/12/40/prepub

## Supplementary Material

Additional file 1 Search strategy details. Click here for file

Additional file 2: eTable 1 Studies with moderate or high risk of bias according to pre-defined criteria. Click here for file
