# Supplementary material for: Classification of healthcare-associated infection: a systematic review 10 years after the first proposal
Source: BMC Med. 2014 Mar 6;12:40. doi: 10.1186/1741-7015-12-40 (PMC4016612; doi:10.1186/1741-7015-12-40)
Supplement: Additional file 1 — Search strategy details. [file 1741-7015-12-40-S1.docx]

**Additional file 1 – Search strategy details**

**Pubmed query:**

((((“healthcare” [All Fields] OR “health care” [All Fields] OR “health-care” [All Fields]) AND (“related” OR “associated” [All Fields])) OR “healthcare-related” [All Fields] OR “healthcare-associated” [All Fields] OR “health care-related” [All Fields] OR “health care-associated” [All Fields]) AND (“Infection” [MeSH Terms] OR “bacteremia” [MeSH Terms] OR “endotoxemia” [MeSH Terms] OR “bacteraemia” [All Fields] OR “sepsis” [MeSH Terms] OR “severe sepsis” [MeSH Terms] OR “septicemia” [MeSH Terms] OR “pyohemia” [MeSH Terms] OR “pyemia” [MeSH Terms] OR “pyaemia” [MeSH Terms] OR “poisoning, blood” [MeSH Terms] OR “blood poisoning” [MeSH Terms] OR “pneumonia” [MeSH Terms] OR “pneumonia lobat” [MeSH Terms] OR “lobar, pneumonia” [MeSH Terms] OR “pneumonitis” [MeSH Terms] OR “pulmonary inflamation” [MeSH Terms] OR “lung inflamation” [MeSH Terms] OR “bronchopneumonia” [MeSH Terms] OR “peritonitis”[MeSH Terms] OR “cystitis”[ MeSH Terms] OR “pyelonephritis”[ MeSH Terms] OR “pyelocystitis”[ MeSH Terms]) AND “adult”[Filter])

**ISI web of knowledge query**

((("healthcare" OR "health care" OR "health-care") AND ("related" OR "associated")) OR "healthcare-related" OR "healthcare-associated" OR "health care-related" OR "health care-associated") AND ("Infection" OR "bacteremia" OR "endotoxemia" OR "bacteraemia" OR "sepsis" OR "pneumonia" OR "bronchopneumonia" OR "peritonitis" OR "cystitis" OR "pyelonephritis" OR "pyelocystitis")

**SCOPUS query**

(((("healthcare" OR "health care" OR "health-care") AND ("related" OR "associated")) OR "healthcare-related" OR "healthcare-associated" OR "health care-related" OR "health care-associated") AND ("Infection" OR "bacteremia" OR "endotoxemia" OR "bacteraemia" OR "sepsis" OR "pneumonia" OR "bronchopneumonia" OR "peritonitis" OR "cystitis" OR "pyelonephritis" OR "pyelocystitis")) *in* TITLE-ABS-KEY-AUTH
