# Supplementary material for: Classification of healthcare-associated infection: a systematic review 10 years after the first proposal
Source: BMC Med. 2014 Mar 6;12:40. doi: 10.1186/1741-7015-12-40 (PMC4016612; doi:10.1186/1741-7015-12-40)
Supplement: Additional file 2: eTable 1 — Studies with moderate or high risk of bias according to pre-defined criteria. [file 1741-7015-12-40-S2.docx]

**Additional file 2: eTable 1** – Studies with moderate or high risk of bias according to pre-defined criteria

| Study | Defines inclusion criteria | Defines selection method | Consecutive selection of patients | Attrition bias | Reporting bias | Overall |
| --- | --- | --- | --- | --- | --- | --- |
| Rodriguez-Bano [19] | yes | yes | Not mentioned |  | partial | Moderate |
| Umeki [22] | yes | yes | yes | no | partial | Moderate |
| Seki [23] | yes | yes | yes | no | partial | Moderate |
| Park [[9](#_ENREF_9)] | yes | yes | yes | no | partial | Moderate |
| Wu [29] | yes | yes | yes | no | partial | Moderate |
| Al-Hasan[31] | yes | yes | Not mentioned | no | no | Moderate |
| Siegman-Igra[[7](#_ENREF_7)] | yes | yes | yes | partial | no | Moderate |
| Friedman [[3](#_ENREF_3)] | yes | yes | yes | yes | yes | High |
| Evans [17] | yes | yes | Not mentioned | yes | yes | High |
| Vallés [[8](#_ENREF_8)] | yes | yes | yes | yes | yes | High |
| Pascual [21] | yes | yes | yes | yes | yes | High |
| Aguilar-Duran [[6](#_ENREF_6)] | yes | yes | yes | yes | yes | High |
| Chalmers [36] | yes | yes | yes | yes | yes | High |
| Kollef [[13](#_ENREF_13)] | yes | yes | yes | no | yes | High |
| Miyashita [39] | yes | yes | yes | no | yes | High |
| Swenson [43] | yes | yes | no | no | partial | High |
| Kao [30] | yes | yes | yes | no | yes | High |
| Guimarães [35] | yes | yes | yes | no | yes | High |
| Sy [46] | yes | yes | yes | no | yes | High |
